# What effort is required in retrieving self-defining memories? Specific autonomic responses for integrative and non-integrative memories

**DOI:** 10.1371/journal.pone.0226009

**Published:** 2019-12-05

**Authors:** Audrey Lavallee, Xavier Saloppé, Marie-Charlotte Gandolphe, Laurent Ott, Thierry Pham, Jean-Louis Nandrino

**Affiliations:** 1 Cognitive and Affective Sciences Laboratory, Joint Research Center (UMR9193) of the French National Center for Scientific Research, University of Lille, Villeneuve D’Ascq, France; 2 Department of Legal Psychology, University of Mons, Mons, Belgium; 3 Research Center in Social Defense, Tournai, Belgium; 4 Psychiatric Hospital, Saint-Amand-les-Eaux, France; University of St Andrews, UNITED KINGDOM

## Abstract

Self-defining memories (SDM) are autobiographical memories associated with the construction and maintenance of identity, and which play a core role in establishing and achieving goals in life. The aim of this study was to evaluate the effort required in retrieving SDM as reflected by physiological activity. We examined the neurovegetative responses associated with three dimensions of SDM: specificity, integrative meaning and emotional valence. Electrodermal activity (skin conductance response frequency, phasic driver) and the high frequency component of heart rate variability (HF-HRV) were recorded during the retrieval of SDM in 36 healthy participants to assess the activation of the sympathetic and parasympathetic systems, respectively. SDM were characterized by three independent investigators with satisfactory inter-rater reliability. Linear mixed effects analyses showed that only the integrative meaning dimension led to a main effect on electrodermal activity. In addition, an interaction effect showed that the retrieval of non-integrative and specific memories was associated with a higher level of electrodermal activity than the retrieval of integrative specific memories. No effects were obtained regarding the HRV indicators. The higher activation of the sympathetic nervous system associated with the retrieval of non-integrative SDM suggests that the ability to derive meaning from personal experiences plays a regulatory role and is a fundamental component in personal adjustment.

## Introduction

Autobiographical memory (AM) is the memory of personal experiences and facts about the self [1,2]. It encompasses knowledge about ourselves and our life at different levels ranging from general knowledge to highly contextual-specific personal events [1,3,4]. AM can be seen as a summary of past experiences in preparation for the future [5]. A core characteristic of AM is its uneven temporal distribution with three distinct periods (childhood amnesia, reminiscence bump, and recent events) [3,6]. The reminiscence bump is marked by a substantial rise in memories of events that occurred between the ages of 10 and 30 years. According to Rubin, Rahhal and Poon, the reminiscence bump is a critical period in the construction of identity, notably because it is coincides with some important events in life (e.g.: first amorous relationship, first job, first diploma) [7]. Two distinct periods may be observed within the reminiscence bump. During adolescence, people construct their social identity (integration in society, in group membership); during early adulthood, they develop intimate close relationships [8,9]. Before adolescence, memories are less integrated and the self-schemas of the long-term self are not available yet [10]. Hence, the reminiscence bump is a period in which specific events generate self-defining memories (SDM), although such events may occur at any time in life [3,11,12]. SDM are vivid, emotionally intense and repetitive memories that concern lasting issues or unresolved conflicts [13]. They help maintain self-coherence and self-consistency, especially during periods of difficult transitions or tribulations [14]. Research has highlighted the role of SDM in imagining important future events [15], the development of personality [16], the elaboration and the pursuit of long-term goals [5], the activation of specific self-concepts [17], and emotion regulation [16,18,19]. Because SDM are emotionally intense they are frequently share with others to favor close relationship and the transmission of personal experience[20,21]. In addition SDM play a core role in the construction of life goals and are considered to provide more insights and understanding of the meaning of life than other personal memories [21]. SDM facilitate personal growth thanks to the conception of life’s goals that are coherent with the self [22]. Furthermore, the ability to attribute meaning to the past events recalled in SDM is associated with optimal levels of adjustment, self-restraint and emotional expression [14,22,23].

Singer and Blagov proposed an SDM classification system based on their specificity, integrative meaning [24,25], and affective valence [26]. Their level of specificity depends on the duration, the onset, and the level of sensory details of the event recalled. Integrative meaning refers to the ability to learn lessons about events contained in the SDM. Finally, the affective dimension corresponds to the valence of SDM as expressed in the discourse of individuals.

While the literature on SDM provides a clear-cut identification of their characteristics and their changes according to age or pathology [27–29], no study, to our knowledge, has measured the physiological activation related to the retrieval of SDM. This would provide an objective measure of the emotional and cognitive load of SDM. Schaefer and Philippot previously used physiological markers to study emotion activation during the retrieval of AM, which were experimentally constrained by their affective valence (positive, negative and neutral) [30]. In their study, they measured the cardiac inter-beat interval and the mean level of electrodermal activity, which both indicate activation of the sympathetic nervous system. Whatever their valence, autobiographical emotional memories elicited higher physiological responses than neutral memories. However, the study by Schaefer and Philippot did not evaluate the physiological responses associated with other characteristics of AM (e.g. specificity, integrative meaning) and did not focus on SDM. As already demonstrated by Singer and Moffitt, when the individuals retrieved SDMs–unlike AMs—they subjectively considered them more emotionally intense, more important and as having a content directly associated with self-construction, so we think that recall of SDMs acts as a physiological activator [31].

The present study investigated the processes underlying the retrieval of SDM by evaluating whether according to their dimensions of specificity, affective valence and integrative meaning, they require a specific effort, as reflected by electrodermal activity (EDA) and heart rate variability (HRV) during their recall. These markers reflect the activity of the sympathetic and parasympathetic nervous systems, respectively, and are classically used to characterize cognitive and affective processes [32–36]. Our research question was to examine whether each SDM’s characteristic was associated with a particular physiological signature and thus to characterize its potential emotional and cognitive cost.

## Method

### Participants

Participants were recruited through an advertisement on a social network inviting them to take part in a study on AM. Forty healthy participants were recruited. Two were excluded because they retrieved only four memories, another for misunderstanding the instructions and a fourth due to a technical problem with the Biopac MP150®. The final sample thus consisted of 36 healthy participants with a mean age of 26.83years (SD = 2.73; 18 men and 18 women) and a higher education level (at least two years after high school diploma). A clinical interview (based on Mini’s dimensions[37]) with a psychologist established that none of them had any serious psychological disorders or somatic illnesses. They were asked not to consume caffeine or tobacco in the hours preceding the experiment and were all debriefed after the study.

### Compliance with ethical standards

The ethical committee of Les Marronniers Hospital in Tournai (Belgium) gave approval for the present study to be conducted (reference CE/DV/EA/2015). The procedure was also conducted in accordance with the 1964 Helsinki Declaration and its later amendments or comparable ethical standards. All participants were recruited on a voluntary basis. Participants were recruited through an advertisement disseminated on social networks and among the students at the University of Lille in France and UMons in Belgium. After the participant contacted an investigator, he/she received an email containing an information letter, the instructions of the study, information on how to go to the meeting location and several possible appointment dates. Before starting the experimental procedure, the goal and the instructions of the study were again explained to the participants by the investigator in order to ensure they had a clear understanding of the procedure. Then they signed two consent forms, one kept by the investigator and the other by the participants and in which their personal identifier code was indicated. Thanks to their identifier code, they can had access to their personal information and could request it to be destroyed. No one asked the destruction of his/her personal data.

### Experimental procedure

Participants first completed a questionnaire assessing their height, weight, menstrual cycle period, dominant hand, tobacco and caffeine consumption, and psychiatric and medical history. Next, electrodes for physiological recording were attached to the participants and they were asked to avoid moving. They were then asked to recall five SDM as follows: “We would like you to remember five events in your life. These events must be important in defining who you are. In other words, these memories should refer to events that help you to understand who you are as an individual. They should also be events that you would share with someone if you wanted that person to understand you in a basic way. The events may be positive or negative memories. The only important aspect is that they should lead to strong feelings. The memories should be events that you have thought about many times. They should also be familiar to you like a picture you have looked at a lot or a song you learned by heart”. These instructions replicated those used in previous SDM research [31]. During the narration of an SDM, the experimenter was seated in front of the participant and did not interact with him/her. After each SDM, the participant was debriefed and allowed to relax in order to return to his/her physiological baseline.

### Coding of self-defining memories

SDM were recorded by means of a microphone throughout the experiment and were transcribed and evaluated a posteriori using Singer and Blagov’s classification system and scoring manual for self-defining autobiographical memories [24,25]. According to this classification system, SDM were coded as specific if they were a memory of a specific brief event with a unique occurrence with perceptual and sensory details. In other words, an SDM is a delineated memory in which an event has an identifiable beginning and end and does not exceed 24 hours in which the individuals describe their feelings. (e.g.: « When I defended the thesis of my master’s degree on Tuesday, June 16, 2016, it went pretty well and I received many compliments. I was very proud of myself »). They were coded as general when they referred to a memory of long or repeated events [24,25] (e.g.: « My week’s vacation in the Pyrenees » or « Every year in July I went to my grandmother’s house on the seashore »). Exceptions were considered for categorizing specific memories: if memories seemed general (with lots of contextualization details) but included a specific event associated with details and the expression of emotion, they were coded as specific (e.g.: « When I was young we usually went fishing with my parents, and now I’m happy to reproduce that with my own children. I remember when my younger son has caught his first fish. It was an amazing moment. ». In addition, if memories seemed general but contained a speech or dialogue with details about a person, these were also coded as specific. If the events lasted for more than 24 hours but corresponded to a memory of a specific event, then the memories were also coded as specific (e.g.: «The birth of my first daughter was very long, I felt my first real contractions on Sunday, October 5, 2014 about noon and I gave birth Wednesday night »).

Concerning the affective valence dimension, memories were coded as positive, negative, neutral or mixed according to the emotional words used and whatever the theme retrieved. If no emotional words were expressed, the memory was categorized as neutral (e.g. « After my parents got divorced, we spent alternate weeks with each parent until I was 18. »). If only positive emotional words were expressed, the memory was categorized as positive and if only negative emotional words were used, as negative. If the memory contained both positive and negative emotional vocabulary, it was coded as a mixed memory (e.g. « It is a pleasant memory today but when it happened it was very embarrassing. ») [26].

In agreement with the classification system of Singer and Blagov and concerning the integrative meaning dimension, memories were considered “integrated” if they corresponded to a value or a meaning that the individual derived from his/her experience (e.g. « It is through that experience I have discovered I was passionate by undersea life and the importance of protecting it.»). Two types of integrative meaning were distinguished: meaning directly linked to the self and meaning linked to others or the environment. SDM were considered “non-integrated” if they were pure narrative not clearly related to learning about oneself, others or the environment [24,25].

Three independent investigators categorized the SDM. For each dimension, the Cohen-Kappa coefficient was calculated to measure inter-rater agreement. The coefficients were all satisfactory and ranged from substantial to almost perfect agreement (specificity: k = 0.79; affect: k = 0.87; integrative meaning: k = 0.80).

### Physiological acquisition and signal analysis

A physiological Biopac MP150® amplifier was used for physiological signal acquisition. For electrocardiogram recording (ECG), two EL503 pre-gelled electrodes were placed on the participants’ wrists. The parasympathetic nervous system activity involved in regulation processes was specifically measured by analyzing high-frequency changes in the heart inter-beat interval (HF-[0.15; 0.40Hz]-) [38]. To take intra- and inter-individual variability into account, HF-HRV was natural-log transformed for statistical analyses. Finally, to compute the high frequency component of the HRV reliably, a minimum of 1 minute of ECG recording is needed so only SDM lasting one minute or more were selected for the statistical analysis of the HRV.

Concerning EDA recording, the ambient temperature was maintained at a temperature around 21–23 degrees Celsius. Two electrodes were placed on the distal phalange of the index and middle fingers of the non-dominant hand. The electrolytic mixture GEL 101 was applied inside the electrodes and on the distal phalanges. EDA variability corresponds to the variation in the secretion of the eccrine glands, which are innervated exclusively by the sympathetic nervous system [39]. The skin conductance response (SCR) corresponds to an increase with a minimum amplitude criterion of 0.05μS in comparison to the skin conductance tonic level, driven by the impulse of the sympathetic nervous system on the eccrine glands. Since the signal is recorded over a long and variable period, the frequency of the SCR (SCR frequency) was computed. A continuous decomposition analysis was also carried out to evaluate the phasic driver (PD), which corresponds to the phasic component of the signal (phasic driver = total driver signal–tonic driver) (for review see [40,41]).

### Statistical analysis

Statistical analyses were performed with R version 3.5.1. SDM were characterized according to specificity, affective valence and integrative meaning. Chi-squared tests were applied. To investigate whether the specificity, valence and integrative meaning dimensions of SDM influenced physiological activation, and because each participant provided five SDM, linear mixed-effects models (lme4 1.1–19 package, [42]) were conducted for the electrodermal activity indicators (SCR frequency and mean phasic driver) and for the marker of cardiac activation (HF-HRV). The independent variables were the Participants [n1; n36] (nominal random factor), Specificity with two categories {Specific, General} (dichotomous fixe factor), Valence with four categories {Positive, Negative, Neutral, Mixed} (polytomous fixe factor) and Meaning with two categories {Integrative, Non-integrative} (dichotomous fixe factor). The General, Neutral and Integrative categories were used as reference for statistical analyses. The dependent variables of the EDA corresponded to the markers of sympathetic nervous system activation: the Phasic Driver and the Skin Conductance Responses frequency per minute (SCR frequency). For cardiac activity, the dependent variables were the logarithmic transformation of the High Frequency of the Heart Rate Variability (HF-HRVlog). As our models included a participant level random effect on the intercept, the fixed effects were estimated relative to the baseline of the signal of interest taken at the reference categories for each participant. Thus, it seemed unnecessary to apply a baseline correction prior to the analysis.

After studying the main effects of the different factors on the physiological variables, we also examined the interaction effects of the factors on the dependent variables. For each model, a chi-squared test was done on the likelihood ratio. When necessary, post-hoc tests were conducted (lsmeans, 2.30–0 package, [43]).

The models calculating main effects were labeled with the initials ME followed by the initials of the dependent variable. Similarly, the models calculating interaction effects were labeled with the initials IE followed by those of the dependent variable. Models were described as follows:

ME_PD = Phasic driver ~ (specificity + valence+ meaning + (1|participant))

ME_SCR = SCR frequency ~ (specificity + valence+ meaning + (1|participant))

ME_HRV = HF-HRVlog ~ (specificity + valence + meaning + (1|participant))

IE_PD = Phasic driver ~ (specificity * valence + specificity * meaning + valence * meaning + (1|participant))

IE_SCR = SCR frequency ~ (specificity * valence+ specificity * meaning + valence * meaning + (1|participant))

IE_HRV = HF-HRVlog ~ (specificity * valence + specificity * meaning + valence * meaning + (1|participant)).

The level of significance was 0.05 and, when necessary, effect sizes were computed using the adapted Cohen’s d linear mixed-effect model and Westfall’s d [44–46].

## Results

Thirty-six participants retrieved 5 SDM, giving a total of 180 SDM. For technical reasons the HRV analyses could not be used for 3 SDM, and among the remaining 177, only 149 SDM lasted one minute or more so these were used for the HRV analyses.

### Characteristics of self-defining memories

According to the specificity criteria, most of the SDM were specific (67,22%) and 32.78% were general (χ^2^ = 21.36, *p* < .001). On the affect dimension, 31.11% of the SDM were mixed, 30.00% were negative, 22.78% were positive and, 16.11% were neutral (χ^2^ = 10.53, *p* = .015). Regarding integrative meaning, 45% of the memories were integrative and 55% were non-integrative (χ^2^ = 1.8, *p* = .179). The average duration of the SDM was 2.67min (SD = 2.30 min).

### Main and interaction effects of physiological markers according to characteristics of self-defining memories

**Fig 1:**
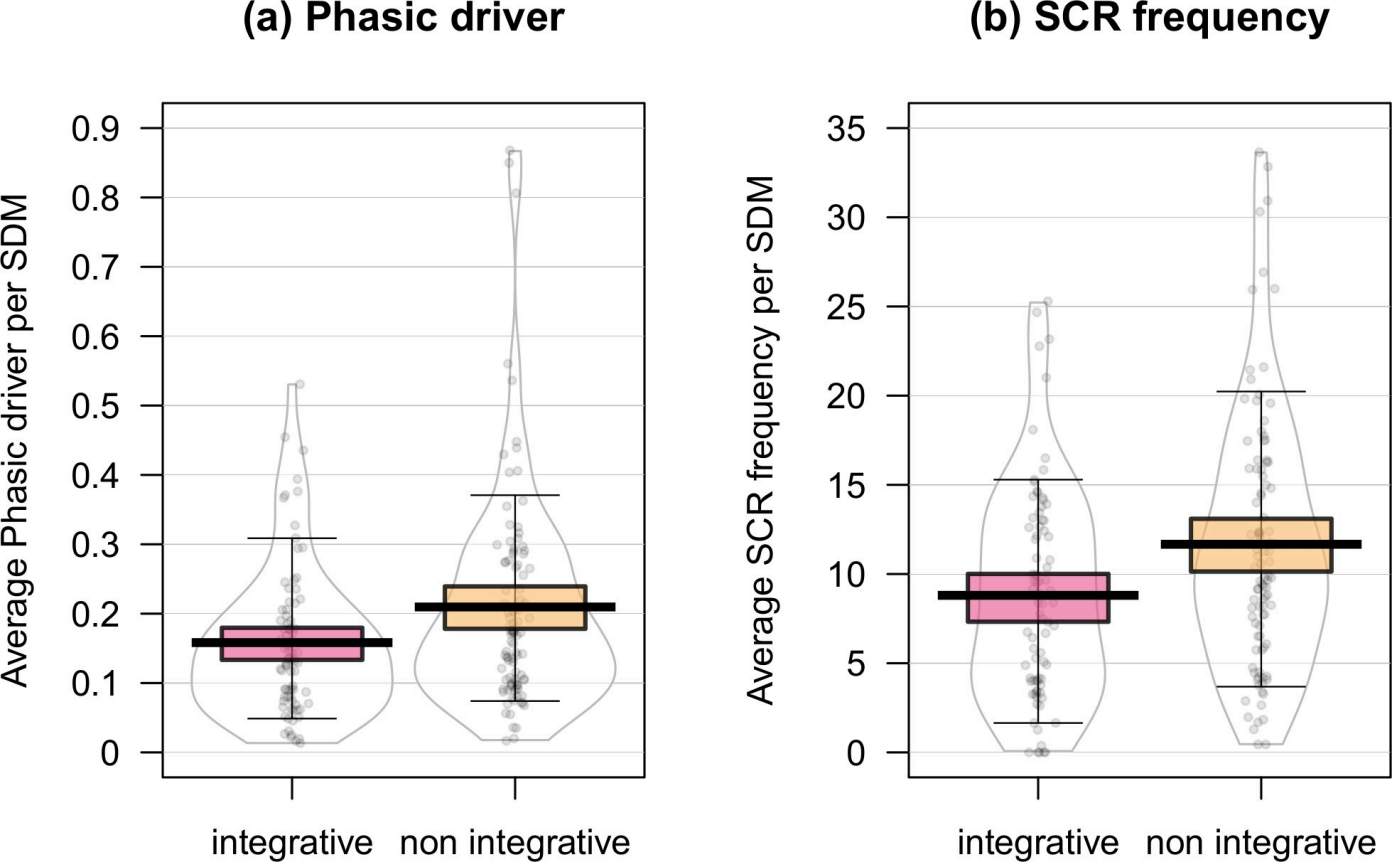
Comparisons of physiological responses in integrated and non-integrated SDM. Pirateplot (median, interquartile and data spread) (Yarrr, 0.1.5 package,[47]) of phasic driver (a) and SCR frequency (b) within integrative and non-integrative SDM. The units of measurement are μS for the phasic driver dependent variable and the average number of skin conductance responses per minute for the SCR frequency dependent variable.

Concerning electrodermal activity (EDA, models ME_PD and ME_SCR), no main effect was observed for specificity, valence factors on the phasic driver (Specificity: LRT = 1.43, *p =* .231; Valence: LRT = 1.33, *p =* .722), or on SCR frequency (Specificity: LRT = 0.32, *p =* .573; Valence: LRT = 1.94, *p =* .584) variables. Statistical analyses revealed a main effect of integrative meaning on the phasic driver (Estimate = 0.04, SE = 0.01, LRT = 6.38, *p =* .012, Westfall’s d = .32) and on SCR frequency (Estimate = 1.67, SE = 0.69, LRT = 5.85, *p =* .016, Westfall’s d = .25). The mean of the phasic driver was 0.16μS (SE = 0.02) for integrative memories and 0.20μS (SE = 0.02) for non-integrative ones. The mean number of electrodermal responses per minute was 9.46 (SE = 1.06) for integrative memories and 11.13 (SE = 1.05) for non-integrative ones. Results are illustrated in Fig 1.

An interaction effect was observed between specificity and integrative meaning factors on SCR frequency (model IE_SCR) (LRT = 6.68, *p =* .009). The mean number of electrodermal responses per minute was 8.99 (SE = 1.14) for specific and integrative memories and 11.84 (SE = 1.07) for specific and non-integrative memories (Estimate = 3.44, SE = 1.31, T = 2.62, *p =* .002, Westfall’s d = .42). No interaction effect was observed between integrative meaning and the valence factors (LRT = 0.24, *p =* .971), nor between specificity and the valence factors (LRT = 7.25, *p =* .064) on the SCR frequency variable. No interaction effect was observed between specificity and the integrative meaning factors (LRT = 2.69, *p =* .101), between valence and the integrative meaning factors (LRT = 1.75, *p =* .627), or between specificity and the valence factors (LRT = 5.04, *p =* .169) on the phasic driver variable (model IE_PD).

Concerning HRV (model ME_HRV), no main effect was observed for specificity (LRT = 0.48, *p* = .539), affect (LRT = 5.70, *p* = .127) and meaning (LRT = 0.37, *p =* .541) factors on the HF-HRVlog variable.

## Discussion

In this study examining whether the characteristics of specificity, affective valence and integrative meaning of the SDM activate the autonomous nervous system, most SDM were specific (67.22%), corresponding to the level generally observed in healthy populations [5,31,48]. Concerning the affective valence dimension assessed by an objective measure of emotional words used, most of the SDM recalled were mixed (31.11%; positive: 22.78%; negative: 30.00%; neutral: 16.11%). According to Wood and Conway, this high frequency of mixed memories in healthy people corresponds to the natural evolution of memories in order to maintain self-esteem and coherence in the sense of identity [19]. Indeed, the affective valence of the memories is transformed over time to become both negative and positive. This transformation allows individuals to master their problem-solving strategy skills providing them confidence about their own abilities. It allows also to enhance the positive affect associated with the event in order to preserve the illusion of a bright future, to maintain self-esteem, well-being and good mental health [3,19,49]. Finally, regarding the meaning dimension, there were no significant differences between integrative and non-integrative SDM (45% and 55%, respectively). This is consistent with the study by Blagov and Singer, in which participants recalled almost 30% of integrative and 70% of non-integrative SDM [23].

Regarding activation of the autonomous nervous system, there was an increase in EDA markers on the integrative meaning dimension and in the interaction between specificity and the integrative meaning dimensions. However, this was the case only for activation of the sympathetic nervous system but not for the parasympathetic nervous system (HF-HRV; ME_HRV model). Non-integrative memories led to a higher activation of the sympathetic nervous system, as shown by the amplitude of the phasic driver (ME-PD model) and by the number of electrodermal responses per minute (ME_SCR) in comparison with integrative memories. This suggests that when memories are not integrated, the level of activation increases. Since one of the main functions of autobiographical memories, and notably of the SDM, is to establish and achieve life goals (for review see [14,21]), autobiographical memories may serve to recall how life goals were established and achieved in the past. Integrative memories are memories that have been used to achieve past goals, while memories without an explicit meaning might concern goals in progress or aborted projects. A potential explanation of the activation of the sympathetic response could be the reliving of events and perhaps the emotional load associated with the retrieval of memories. The emotional dimension of memories could be seen as the centerpiece of the motivation for change and to reduce the discrepancy associated with the creation of life goals [3]. In the study by Çili and Stopa, individuals retrieving SDM used more emotional states to describe themselves in non-integrative memories than in integrative memories [17]. The emotional load of integrative memories might thus be regulated; leading to lower activation of the sympathetic nervous system than with non-integrative memories.

Furthermore, statistical analyses highlighted an interaction effect between specificity and integrative meaning on the activation of the sympathetic nervous system (IE_SCR). Specific non-integrative SDM led to higher activation of the sympathetic nervous system than did specific integrative memories. Interestingly, this activation of the sympathetic nervous system was observed only for the specific memories, which correspond to the recall of single brief events containing spatiotemporal, perceptive and emotional details. According to Conway, Singer and Tagini, the ability to retrieve objectives in a precise and detailed way might allow the pursuit of new goals and avoid repeating experiments [14]. The details in specific memories might provide better understanding of how past events occurred, so that future objectives might be more easily achieved and memories more easily integrated [50].

On the other hand, no significant variations in physiological responses were observed on the valence dimension of SDM. As in the study by Schaefer and Philippot, neutral memories were used as a reference for statistical analyses [30]. However, we did not find any differences between neutral memories and emotional ones. This absence of variation could be due to the nature of SDM, which are highly emotional. Finally, Blagov and Singer [23] found that the integrative meaning, specificity of SDM were largely independent of their valence dimensions, which may account for the absence of interaction effects observed between these variables in our study.

In addition, we explored whether the retrieval of SDM would involve a variation in cognitive load according to their type, which would have led to a variation in HRV. HRV is a sensitive marker of cognitive processes, their level decreasing as cognitive load increases [32,51]. As no variation was observed in terms of the characteristics of SDM, we cannot attest that the type of SDM affected variations in the parasympathetic nervous system and regulated activity associated with retrieval.

This study has some limitations, especially regarding the personality of the participants. Although we controlled for personality disorder, we did not evaluate the level of personality functioning (i.e. extraversion, neuroticism). Blagov and Singer [23] showed that the number of integrative memories recalled varied according to self-reported scores on the Weinberger Adjustment Inventory scale (WAI; [52]). Individuals with a moderate level of self-restraint–which corresponds to the ability to control emotions and impulses- retrieved more integrative memories than those with low (control deficit) or high levels (inflexibility and over- control). Future studies should include personality functioning variables in order to explain potential physiological variations during the retrieval of SDM. Another limitation concerns the age of the participants, who were in the middle of the reminiscence bump and thus building their personal identity. Singer, Rexhaj and Baddeley (2007) found that persons aged 50 years and older retrieved more positive affective valence memories that contained more integrative meaning than college students did [53]. In future study, it would be interesting to replicate the present study with older people to investigate whether non-integrative memories maintain their physiological activation across life. Finally, to examine whether SDM trigger physiological activation compared to other autobiographical memories, it would be interesting in future study to replicate this experimental design with both types of memory retrieval.

Nevertheless, the present study provides better understanding of the physiological processes associated with SDM and particularly with the integrative meaning dimension. The retrieval of non-integrative SDM leads to activation of the sympathetic nervous system, which may be associated with emotional non-regulated content.

## Supporting information

S1 Supporting information Information questionnaire. (PDF)

S1 Dataset (7Z)
