# Supplementary material for: What effort is required in retrieving self-defining memories? Specific autonomic responses for integrative and non-integrative memories
Source: PLoS One. 2019 Dec 5;14(12):e0226009. doi: 10.1371/journal.pone.0226009 (PMC6894867; doi:10.1371/journal.pone.0226009)
Supplement: S1 Supporting information — (PDF) [file pone.0226009.s001.pdf]

Audrey Lavallée
Pr Thierry Pham
Pr Jean-Louis Nandrino
Equipe Dynamique Émotionelle et Pathologie
Laboratoire SCALab UMR CNR S 9193
Université de Lille

Partie à compléter par l'expérimentateur :

T° : *Celsius* Heure :

Date :

Code Participant :

1. Age : ans
2. Poids : kg
3. Taille : cm
4. Sexe : Homme ☐ Femme ☐
5. Heure du réveil :
6. Fumeur : oui ☐ non ☐
7. Situation familiale :

   Célibataire ☐ En couple ☐ Séparé / Divorcé ☐ Veuvage ☐
8. Nombre d'enfant :
9. Niveau d'étude :
10. Situation professionnelle

    Salarié_CDI ☐ Salarié_CDD ☐ Sans-emploi/chômage ☐

    Au foyer ☐ Etudiant ☐ Invalide/AAH/ Longue maladie ☐
11. Etes-vous : Droitier ☐ Gaucher ☐
12. Etes-vous, avez-vous déjà été sujet à des troubles neurologiques (AVC ; épilepsie …) ? ……
13. Etes-vous, avez-vous déjà été sujet à des troubles psychiatriques ? …………………
14. Suivez-vous actuellement un traitement ? ………………

    Si oui, le(s)quel(s) ?

    ……………………………………………………………………………………………

    ……………………………………………………………………………………………
15. Si vous êtes une femme, prenez-vous des hormones contraceptives ? ……………

    Pouvez-vous nous indiquez la période du cycle menstruel dans laquelle vous vous trouvez?

    ……………………………………………………………………………………………

L'utilisation et la diffusion de ce questionnaire sont interdites sans l'autorisation des auteurs

Audrey Lavallée
Pr Thierry Pham
Pr Jean-Louis Nandrino
Team Dynamique Émotionelle et Pathologie
Laboratory SCALab UMR CNR S 9193
University of Lille

To be fulfilled by the investigator:

Temperature: *Celsius* Hour :

Date:

Participant code:

1. Age: years
2. Weight: kg
3. Height: cm
4. Sex : Man ☐ Woman ☐
5. Wake-up time today:
6. Smoker: Yes ☐ No ☐
7. Family status :

   Single ☐ Couple ☐ Separated/Divorced ☐ Widow(er) ☐
8. Number of children:
9. What is your education level:
10. Professional status :

    Permanent contract employee ☐ Fixed-term contract employee ☐ Unemployed ☐

    Homemaker ☐ Student ☐ Invalid/Handicap ☐
11. You are: Right-handed ☐ Left-handed ☐
12. Have you ever had any neurological disorders (stroke; epilepsy …)? ……
13. Have you ever had any psychiatric disorders? ………………….
14. Are you currently under treatment/on medication? ………………

    If yes, which one(s)?

    ……………………………………………………………………………………………

    ……………………………………………………………………………………………
15. If you are a woman, are you taking contraceptive hormones? …………….

    Could you indicate what period of of the mentrual cycle you are in?

    ……………………………………………………………………………………………

The use and the dissemination of this questionnaire is prohibited without the permission of the authors
